# ASO Author Reflections: Validation Based on Low-Volume Metastasis of the Sentinel Lymph Node Biopsy in Early-Stage Cervical Cancer

**DOI:** 10.1245/s10434-020-09353-1

**Published:** 2020-11-20

**Authors:** Benedetta Guani, Patrice Mathevet

**Affiliations:** 1 Department of Gynecology, CHUV, Lausanne, Switzerland; 2 Faculty of Medicine, UNIL, Lausanne, Switzerland

## Past

Sentinel lymph nodes normally are examined with ultra-staging, and non-sentinel nodes usually are examined only with routine technique. Low-volume metastasis (micrometastasis and isolated tumor cells) can be missed by classical pathologic analysis. For ability to consider the sentinel node technique as safe, clinicians must be sure that its negative predictive value (NPV) is 100%.

## Present

To validate the sentinel lymph node technique from the low-volume metastasis point of view, the “Histopathological validation of sentinel lymph nodes study”,^1^ currently in press, included an ultra-staging examination of all nodes, sentinel nodes, and non-sentinel nodes of all Senticol 1 patients.^2^ To date, the discovery of these low-volume metastases can imply therapeutic decisions that can modify the patient’s management even if their clinical significance remains uncertain. This study^1^ can confirm that the NPV is 100% for optimal mapping with bilateral detection, which also is the low-volume metastasis point of view.

## Future

The sentinel node technique is a safe technique. The future lies in standard use of the sentinel node technique for early-stage cervical cancer patients instead of lymphadenectomy. Clinicians are awaiting the results of Senticol 3^3^ for definitive validation of this technique.
